# Analysis of mass transport in ionic liquids: a rotating disk electrode approach

**DOI:** 10.1038/s41598-020-70301-w

**Published:** 2020-08-10

**Authors:** Andrea Giaccherini, Maher Al Khatib, Serena Cinotti, Emanuele Piciollo, Enrico Berretti, Paolo Giusti, Massimo Innocenti, Giordano Montegrossi, Alessandro Lavacchi

**Affiliations:** 1 Dipartimento di Ingegneria Industriale, Università Degli Studi di Firenze, Via Santa Marta 3, 50139 Firenze, Italy; 2 Dipartimento di Chimica, Università Degli Studi di Firenze, Via della Lastruccia 3, 50019 Sesto Fiorentino, FI Italy; 3 Dipartimento di Biotecnologie, Chimica e Farmacia, Università degli Studi di Siena, Via Aldo Moro 2, 53100 Siena, Italy; 4 LEM s.r.l., Via Leo Valiani, 55/59, 52025 Levane Bucine, AR Italy; 5 CNR, Istituto per la Chimica dei Composti Organometallici (ICCOM), Via Madonna del Piano 10, 50019 Sesto Fiorentino, FI Italy; 6 CDR S.R.L., Via degli Artigiani, 6, 50055 Ginestra Fiorentina, FI Italy; 7 CNR, Istituto di Geoscienze e Georisorse (IGG), Via La Pira 4, 50121 Firenze, FI Italy

**Keywords:** Chemical engineering, Electrochemistry

## Abstract

Ionic Liquids are a promising alternative to water electrolytes for the electrodeposition of metals. These solvents have a much larger electrochemical window than water that expands the potential of electrodeposition. However, mass transport in Ionic Liquids is slow. The slow mass transport dramatically affects the rate of reactions at the solid–liquid interface, hampering the exploitation of Ionic Liquids in high-throughput electrodeposition processes. In this paper, we clarify the origin of such poor mass transport in the diffusion–advection (convection) regime. To determine the extent and the dynamics of the convection boundary layers, we performed Rotating Disk Electrode (RDE) experiments on model reactions along with the finite element simulation. Both the experiments and the finite element modelling showed the occurrence of peaks in the RDE curves even at relatively high rotation rates (up to 2000 rpm). The peak in the RDE is the fingerprint of partial diffusion control that happens for the relative extent of the diffusion and convection boundary layers. In looking for a close match between the experiments and the simulations, we found that the ohmic drop plays a critical role and must be considered in the calculation to find the best match with the experimental data. In the end, we have shown that the combined approach consisting of RDE experiments and finite elements modelling providing a tool to unravel of the structure of the diffusion and convection boundary layers both in dynamic and stationary conditions.

## Introduction

Since its infancy the research on ILs (Ionic Liquids) has been devoted to developing new and green processes in a wide range of application fields, spanning from synthesis^1, 2^ to alternative growth of functional coatings (metals, alloys and semiconductors^3–23^) and even as diathermic fluid^24^ or as media for absorption of pollutants^25–28^. However, despite this research effort, ILs have not yet been exploited on a large scale. Only a few systems are commercially available for extremely specialised niches of application where the interface chemistry is relevant (e.g. electrochemical processes). Many reasons justify the limited exploitation of the research results in commercial systems (e.g. higher corrosion rates^24^ and stability under room condition). Traditional solvents as water are often cheaper and abundant. Moreover, technologies for traditional solvents purification are readily available through large commercial networks. A scale economy of ILs could overcome this economic aspect. Besides, previous researches have raised concerns on ILs stability, along with uncertainties on their environmental impact^29–32^. Indeed, we have no long-lasting operations record, limited knowledge of their effect on the environment, on human health, and about their degradability is still very limited. At the opposite, traditional solvents for industrial processes have a long history (more than a century), and their concerns about environmental impact and working place safety are well understood.

Nevertheless, ILs are preferred to traditional solvents when a different reactivity, smaller vapour pressure or larger electrochemical window is required^6, 9^. Besides, traditional solvents and ILs differ much in their mass transport properties. For instance, viscosity typically ranges around 1 cp, two orders of magnitude lower than most of the ILs^13, 33, 34^. The high viscosity also affects the diffusion coefficient. Indeed the diffusion coefficients in ILs are smaller that in water based electrolytes. This aspect has significant implications in the processes where the rate is controlled by interfacial mass transport of the interface process (e.g. electrochemistry, absorption and heterogeneous catalysis). The poor mass transport dramatically affects electrodeposition. Mass transport limited current may e.g. results in the formation of incoherent coatings (e.g. dendrites) with lack of adhesion and functional properties^35^. The application of forced mass transport (convection) can limit these phenomena. However, controlling convection requires a detailed knowledge of the structure and the evolution of the convection boundary layers in the electrolytes. This subject is mostly uncovered in the literature; a few investigation have been reported mainly addressing the measure of fundamental parameters, such as the dynamic viscosity and the diffusion coefficient^36^.

The present paper explores a methodology based on a hybrid experimental and computational analysis of the RDE (Rotating Disk Electrode) voltammetry as a probe of the concentration profiles in a specific and real case. The RDE provides well-defined mass transport conditions in the convection (diffusion–advection) regimes, allowing the derivation of boundary layers with simple models (Levich equation) and the analysis of boundary layers both in stationary regime. No analytical expression that can adequately describe the dynamics of the voltammetry profile under mixed control of the charge flow (charge transfer and transport) is available at this time^37^. Still, this regime can be described by the discretisation of the convection problem employing finite element analysis. The comparison between experimental and simulated voltammetry, under such mixed control conditions, yield much more information about the system than the analysis of just the limiting current regime. Both experiments and simulations in ILs have been carried out using an exemplary IL, BMImBF_4_ (1-Butyl-3-methylimidazolium tetrafluoroborate), as the electrolyte. This ionic liquid is a promising candidate as an air-stable electrolyte and a greener alternative to cyanide bath for the electrodeposition of noble metals^35^. For the present paper, we selected ferrocene as the electroactive species instead of a noble metal ion, a choice that has been operated because the diffusion of ferrocene in ILs has been intensely studied in the literature delivering a set of well-assessed data for simulation^38, 39^. For the sake of comparison, an analogous approach has been followed for a water-based electrolyte. Numerical modelling of electrochemical processes in water and ILs electrolyte have been already discussed in the literature, and their validity is beyond the scope of the present paper^40, 41^. Here, ferrocyanide has been considered in the simulation instead of ferrocene, for the limited solubility of the latter^42^.

## Materials and methods

### Experimental RDE voltammetry in BMImBF_4_

To run the RDE voltammetry, we used a Parstat 2273 potentiostat together with a manual motor controller (500–2000 rpm). The working electrode was a glassy carbon disk (5 mm diameter) embedded in a PTFE rod (10 mm diameter). High purity BMImBF_4_ (> 99.0%—Sigma Aldrich) with halide content lower than 100 ppm. High purity ferrocene (> 98.0%—Sigma Aldrich) was dissolved in BMImBF_4_ to deliver a 5 mM solution. Every potential in this paper is referred to the Ag/AgCl (KCl sat.) reference electrode. The uncompensated cell resistance between the working and reference electrodes has been assessed before each voltammetry using the current interruption method^43^, and the values of the different measurements were averaged.

### Modelling the RDE voltammetry

The governing equation of the mass transport phenomena is the convection equation:

1 $$\frac{\partial C}{{\partial t}} = D\nabla^{2} C + \mathop v\limits^{ \to } \nabla C$$

where C is the concentration of the chemical species whose transport is described. It has been demonstrated by Levich^44, 45^ that in the case of the RDE, the convection problem reduces to a 1D problem governed by Eq. (2):

2 $$\frac{\partial C}{{\partial t}} = D\frac{{\partial^{2} C}}{{\partial z^{2} }} + v_{z} \frac{\partial C}{{\partial z}}$$

where $v_{z}$ is the component of the velocity field perpendicular to the electrode surface (along the $z$ direction). Von Karmann demonstrated that in the region near the electrode surface an accurate expression for ${v}_{z}$ is Eq. (3)^44, 45^:

3 $$v_{z} = 0.51023 \Omega^{3/2 } v^{ - 1/2} z^{2}$$

The analytical solution of Eq. (2) with the velocity field described by Eq. (3) and with the electroactive concentration set to 0 at the electrode boundary, leads to the famous Levich equation for the limiting current at the RDE (Eq. 4)^44, 45^:

4 $$j_{L} = 0.6208 nFD^{2/3} \Omega^{1/2} v^{ - 1/6} C_{0}$$

where ${j}_{L}$ is the average current density on the electrode at the steady-state, $\beta$ a constant related to the measurement units, $n$ number of electrons involved in the reaction, $F$ the Faraday constant, $D$ the diffusion coefficient of the limiting species, $\Omega$ the rotation rate, $\nu$ kinematic viscosity and ${C}_{0}$ the bulk concentration of the limiting species. According to Levich, the thickness of the diffusive boundary layer is^44, 45^:

5 $$\delta = 1.61D^{{{\raise0.7ex\hbox{$2$} \!\mathord{\left/ {\vphantom {2 3}}\right.\kern-\nulldelimiterspace} \!\lower0.7ex\hbox{$3$}}}} \Omega^{{ - {\raise0.7ex\hbox{$1$} \!\mathord{\left/ {\vphantom {1 2}}\right.\kern-\nulldelimiterspace} \!\lower0.7ex\hbox{$2$}}}} \nu^{{{\raise0.7ex\hbox{$1$} \!\mathord{\left/ {\vphantom {1 6}}\right.\kern-\nulldelimiterspace} \!\lower0.7ex\hbox{$6$}}}}$$

The approximations used to derive the Levich equation only holds^46^ for systems where the Schmidt number (Sc) exceeds 1,000. T. Indeed, for ferrocene in BMImBF_4_ Sc exceeds 10^6^, while for ferricyanide in water it ranges around 2000. However, such analytical form describes only the stationary cases and cannot be used to describe the full voltammetry where dynamic effects such as the appearance of peaks under mixed control of the current occur. The peaks can happen when combinations of the following conditions occur: low rotation rates, high scan rates, high viscosity and small diffusion coefficients, as in the case of ILs. Moreover, when non-equilibrium conditions hold the position and the intensity of the peak depend on the exchange current density and electrolytic ohmic drop. In these cases, a numerical solution of Eq. (1) is required with specific condition concurring to the modelling of the additional phenomena. We can safely model the distribution of the electric field inside the electrolyte as a resistance of 330(3) Ω in series with the working electrode according to the method in section “Experimental RDE voltammetry in BMImBF_4_”). The numerical modelling delves the solution of Eq. (2) when Eq. (3) holds with 2 boundary conditions (at the extremes of the 1D domain) and 1 initial condition while the potential is shifted according to Ohm’s law as elsewhere discussed^47^. The first boundary condition is the Butler-Volmer equation (Eq. 6) on the electrode^48^:

6 $$j_{{\left( {z = 0,t} \right)}} = i_{0} \left( {e^{{\frac{{\beta F\left( {E - E_{0} } \right)}}{RT}}} - e^{{\frac{{ - \left( {1 - \beta } \right)F\left( {E - E_{0} } \right)}}{RT}}} } \right)$$

where ${j}_{0}$ is the exchange current density, $E$ is the potential applied to the electrode, ${E}_{0}$ is the equilibrium potential, and $\beta$ is the electron exchange coefficient^48^. The other boundary condition is:

7 $$C_{{\left( {x = l,t} \right)}} = C_{0}$$

The concentration of the species is constant at the opposite side concerning the electrode (at distance $l$ from the origin). Eventually, the initial condition is:

8 $$C_{{\left( {x,t = 0} \right)}} = C_{0}$$

Representing that at the beginning the reduced species is homogeneously distributed through the whole 1D domain. While $\beta$ can be safely approximated to 0.5 for our purposes, the value of ${j}_{0}$ is hard to measure and not reported in the literature. The values of ${j}_{0}$ and ${E}_{0}$ was optimized exploiting a trial and error approach to reproduce the experimental curve. To this aim, we simulated a set of voltammetries for all the possible combination of ${j}_{0}$ and ${E}_{0}$ respectively in the range between 10^–3^–10 A/m^2^ and 300–600 mV. Then the voltammetry best fitting the experimental data corresponds to the best estimation of ${j}_{0}$ and ${E}_{0}$.

The diffusion coefficient for ferrocene/ferrocinium redox system in BMImBF_4_ it was reckoned using the analysis of the portion of the experimental curves at the steady state. We simulated the ferrocyanide/ferricyanide RDE voltammetry in water the parameter taken as reported in the literature (Table 1). In this case, the resistance of the cell was considered negligible as for highly concentrated supporting electrolyte. We considered the ferrocyanide oxidation as a reversible process setting an extremely high exchange current density. Simulations of the linear sweep voltammetry have been carried out at 2000 rpm rotating rate and a different scan rate: 10 mV/s, 50 mV/s and 100 mV/s and compared with experimental ones. The numerical solutions were obtained using a finite element discretisation of the continuous problem employing the software. Previous researches have shown that Comsol Multiphysics^©^ is an effective tool to simulate dynamic electrochemistry experiments also under convection or on ultramicroelectrodes^49–52^. Table S1 reports more technical details about the computation.

**Table 1:** The values parameters used to run the whole set of calculation presented in this paper.

	${j}_{0}$ (A/m^2^)	${E}_{0}$ (mV)	$D$ (10^–11^ m^2^/s)	$R$ (Ω)	$\nu$ (10^–5^ m^2^/s)
BMImBF_4_	1 10^–1^*	424*	1.75(1)*	330*	9.25^53^
Water	> 10	164^54^	69.5^55^	N/D	0.00829^56^

“BMImBF_4_” refers to the 5 mM solution of ferrocene in BMImBF_4_ and “water” to the 5 mM solution of ferrocynide in water.

*Values reported by this study.

## Results and discussion

### Experimental RDE voltammetry in BMImBF_*4*_

**Figure 1:**
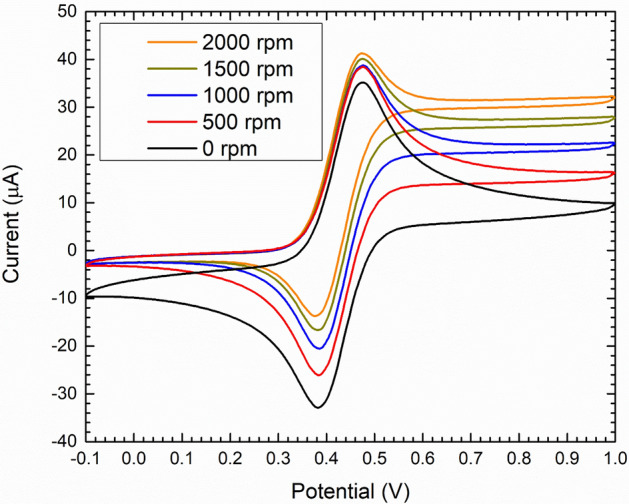
Hydrodynamic cyclic voltammetries of 5 mM ferrocene/ferrocinium in the BMImBF_4_ electrolyte at various rotation rates (scan rate 100 mV/s).

**Figure 2:**
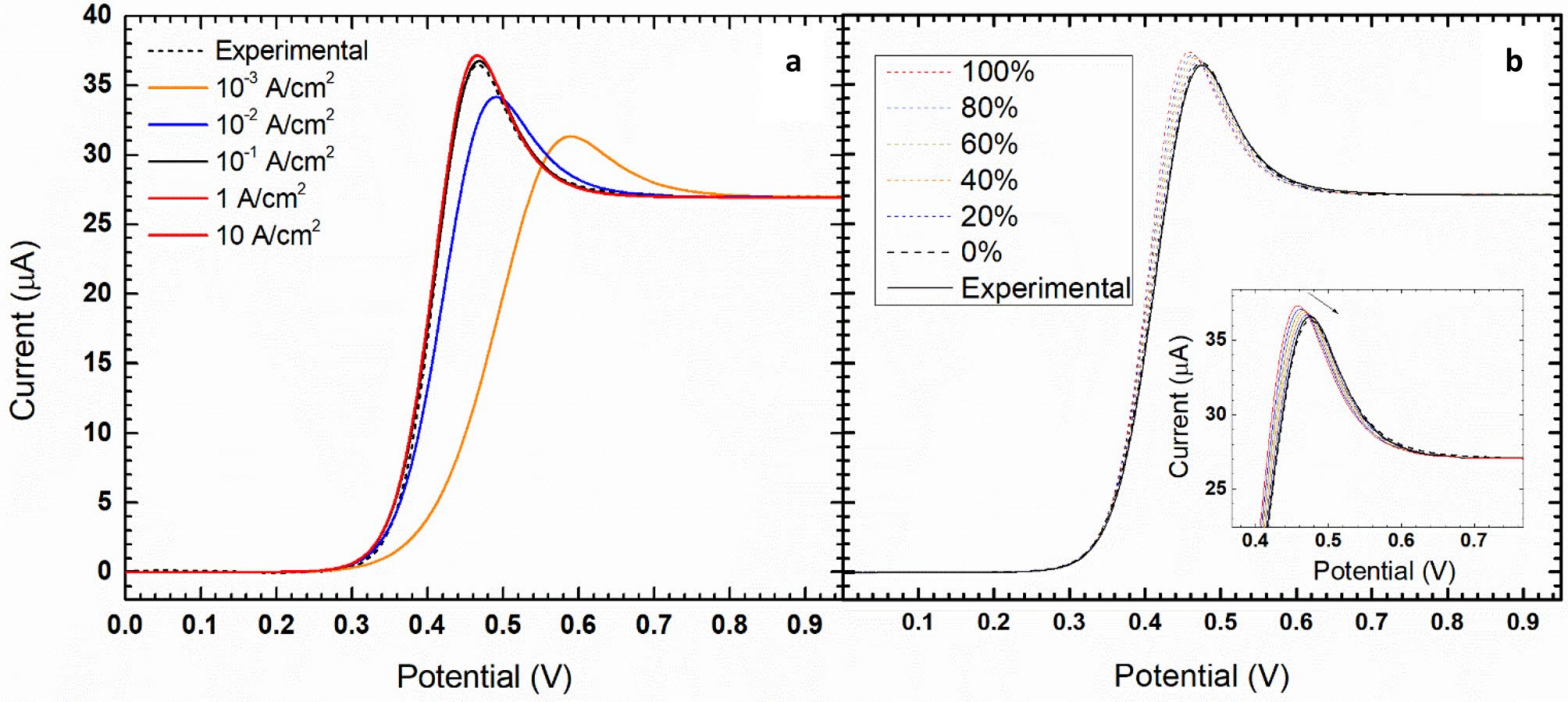
Hydrodynamic voltammetries of 5 mM ferrocene/ferrocinium in the BMImBF_4_ electrolyte at 2000 rpm and 50 mV/s. The discontinuous and continuous lines represent the experimental and simulated curves, respectively. (**a**) The effect of the exchange current density and (**b**) the effect of the compensation on the simulated curves (the arrow marks the decreasing of the compensation).

The RDE voltammetry of ferrocene/ferrocinium in BMImBF_4_ showed a peak up to a rotation rate of 2000 rpm (Fig. 1). Remarkably, water-based electrolytes with the same concentration of the electroactive species and under the same experimental did not show any peak^47^ (ESI Fig. S1). This behaviour results from the lower diffusion coefficient of ferrocene/ferrocinium (1.75 10^–11^ m^2^/s) in the IL compared to the one of ferrocynide/ferricyanide (6.9 10^–10^ m^2^/s) in water and to the large viscosity (9.25 10^–5^ m^2^/s) of BMImBF_4_. Thus, in BMImBF_4_ the convective mass transport is not predominately controlled by advection, and a purely convective regime holds. Furthermore, linear scan voltammetry, recorded at different potential scan rates, show the influence of the mass transport process on the voltammetric profile. As unexpected from the stationary theory of the RDE voltammetry, Figs. 1 and 2 shows a very well-defined peak in the voltammetry at 100 mV/s and a broad peak in the voltammetry at 50 mV/s while the voltammetry at 10 mV/s resembles a standard RDE voltammetry. This peak disappears as the scan rate decreases and the rotation rate increases.

We measured the diffusion coefficient ($D$=1.75(1) 10^–11^ m^2^/s) using the Levich’s law to the linear regression of ${j}_{L}$ plotted against ${\Omega }^{1/2}$ (ESI Fig. S2). Considering the effect of the temperature, not controlled in the RDE experiment (environmental temperature set at 25 °C); this value is in good agreement with those elsewhere reported^38^. The detailed explanation of the reasons for the peak in the RDE voltammetries required a numerical modelling of the experiments. Indeed, no analytical model describe the mixed transport regimes that is responsible for the peak formation in linear sweep voltammetry on RDE.

### Validation of the numerical model

Before proceeding with the simulation of the experiments, we had to validate the numerical model by a comparison of our results with the analytical solution (Levich equation, Eq. 1) for the fully developed advection regime. Table 2 shows the results of the validation both for the IL and water. To avoid the error that the Levich equation produces at low Sc number values, we compared our results with both the Levich’s formula and the more accurate equation reported by Newman in^46^. Considering the Schimdt numbers for the systems investigated here, we found that the use of the Levich’s equation leads to an overestimation of the limiting current of 0.3% in BMImBF_4_ and 3% in water respect to the more accurate Newman’s formula^46^. Most of the discrepancy resides in the approximation needed to calculate the Van Karman's velocity field^46^. The discrepancy occurs because the higher Schimdt number is the more accurate the Levich law (Table 1). Moreover, a comparison with the experimental curves in BMImBF_4_ is needed to validate the model in the non-stationary regime. The validation of the FEA analysis for water-based electrolyte is well discussed in the literature^47^. However, the discrepancy between the analytical solutions (Levich and Newman) and the numerical solution of the RDE problem has to be considered very carefully.

**Table 2:** Results of limiting current densities in the RDE voltammetry at 2000 rpm, experimental values, the result of our simulations, Levich equation all compared with respect to the Newman equation.

	FEA	%	Levich	%	Newman	Exp
BMImBF_4_	27.0 μA	0.3	27.0 μA	0.3	26.9 μA	26.9 μA
Water	688 μA	2.9	688 μA	2.9	669 μA	N.D

The best-estimated values of ${j}_{0}$ and ${E}_{0}$ are reported in Table 1 and Fig. 2a shows that the best fit return 10^–1^ A/m^2^ for ${j}_{0}$. At higher ${j}_{0}$, the shape of the curve become independent on the value of ${j}_{0}$ suggesting that the electron transfer reaction is fast and that these voltammetries show a profile very close to the reversibility. Remarkably, this is in agreement with the symmetric shape of CVs in Fig. 1. On this basis, we can approximate the equilibrium potential as the half-wave potential (${E}_{hw}$ = 0.427 V) and its value is very close to the best fit (${E}_{0}$ = 0.424 V) reported in Table 1.

Figure 2b show the effect of the ohmic drop and its partial and integral compensation on the simulated curves. After the background subtraction, the simulations that best fit with the experimental data are those with no compensation of the cell resistance. We explain this finding considering that the electron transfer kinetics and the correction of the potential due to the uncompensated resistance of the cell define the slope of the curve in the pre-peak range.

**Figure 3:**
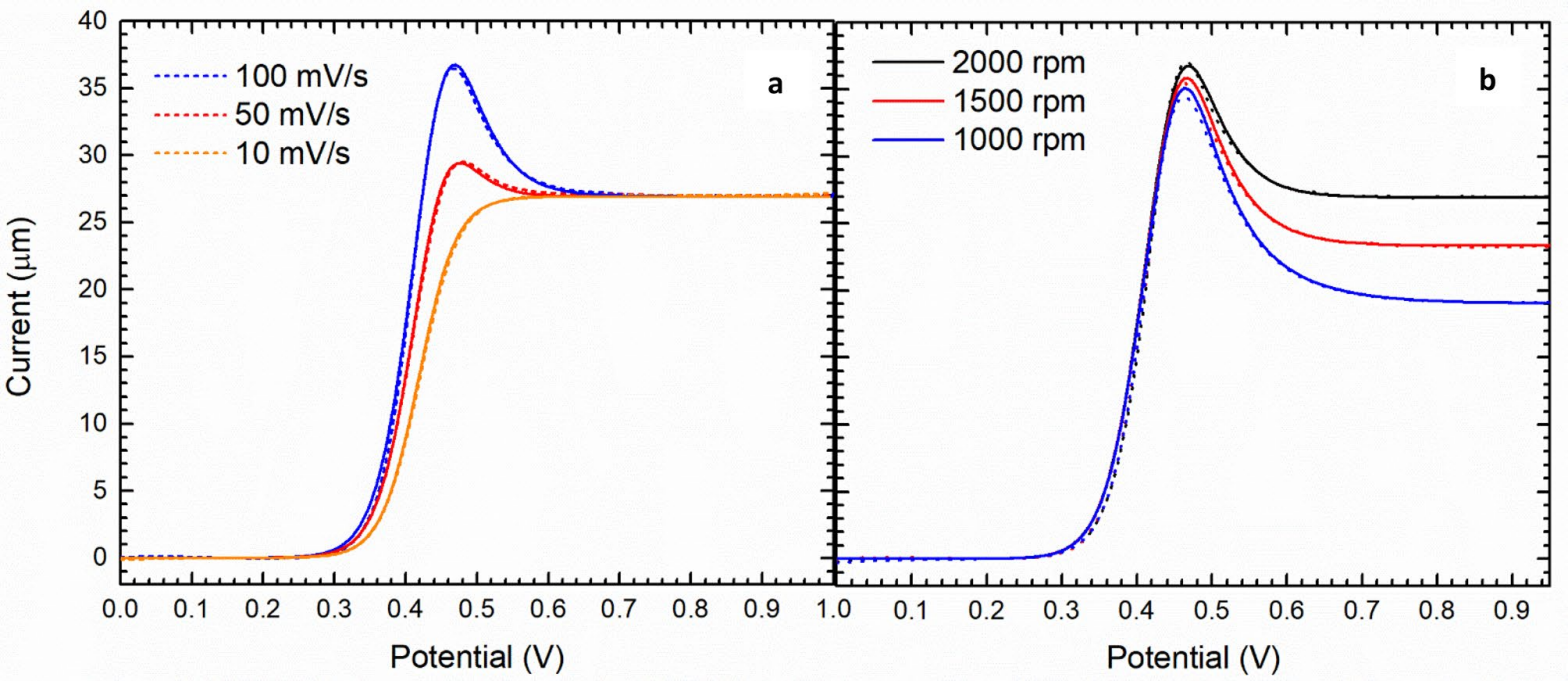
Hydrodynamic voltammetries of 5 mM ferrocene/ferrocinium in the BMImBF_4_ electrolyte. The discontinuous and continuous lines represent the experimental and simulated curves, respectively. The effect of (**a**) scan rate and (**b**) rotation rate.

We have evaluated the quality of the model in the full range of explored scan rate. Figure 3a shows the dependence of the experimental and simulated curves on the scan rate. The peak intensity sharply decreases with the decreasing of the scan rate. Eventually, Fig. 3b shows the dependence of the voltammetric response on the rotation rate showing a good match with the Levich’s law. The peak current decreases with the rotation speed while the width of the peaks increases. The RDE voltammetries simulated with the values reported in Table 1 have a good match with the experimental results (Figs. 2 and 3).

### Study of the convection layers

**Figure 4:**
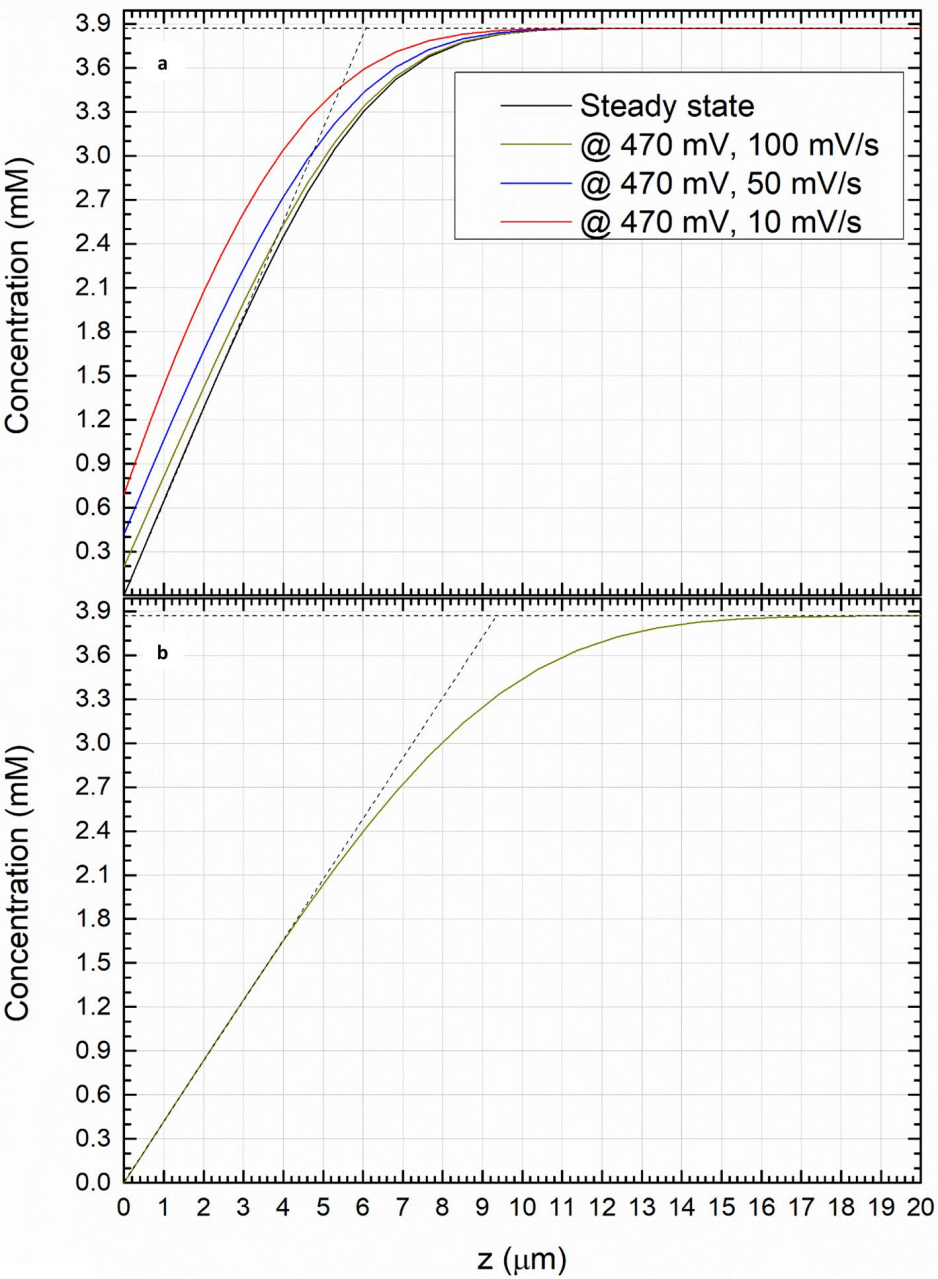
Concentration profiles of the reduced species (Ferrocene for BMImBF_4_ and ferrocynide for water), for all the rotation rates at the steady-state; (**a**) BMImBF_4_ (**b**) water. Dashed lines depict the extrapolation of the convection layer.

Figure 4 shows the concentration profiles, from which the convection boundary layer thicknesses have been extrapolated using the tangent method. In the framework of the Levich theory, the thickness of the convection layer can be assessed (exploiting Eq. 5) as 6.14 μm in BMImBF_4_ and 9.24 μm in water, in good agreement with the numerical results, respectively 6.1 μm and 9.4 μm (Fig. 4). Figure 4a also shows the comparison of the concentration profiles at the peak potential (470 mV) with the one at the steady-state, in BMImBF_4_: at the peak potential (470 mV), these profiles differ according to the different scan rates considered (red, blue and green curves), while they are coincident and superimposed at the steady-state potential (black). It is worth notice that the discrepancy between the concentration profile at the steady-state and the peak potential, increases with the scan rate. Oppositely, the equivalence among all them for ferrocyanide in water is shown in Fig. 4b. Still, the concentration profile at the steady-state is equivalent for each different scan rate, as expected from the Levich theory. The curves in Fig. 4b serve to addresses the presence of the voltammetric peak observed in BMImBF_4_. To understand the fact that at even scan and rotation rates the peak appears in the ionic liquid and not in the water, we start from considering the thickness of the hydrodynamic boundary layers. Qualitatively, the hydrodynamic boundary layer thicker in the ionic liquid than in water, as a result of the different viscosity of the two fluids. Viscosity also affects the diffusion coefficients, determining a slower evolution of the concentration profile in the Ionic Liquid compared to the water (Fig. 4b). Accordingly, we conclude that while in the water convection is effective even at short timescale because there is a significant advection in proximity of the electrode surface, convection is less significant in the ionic liquid. A crude approximation may help in visualizing how this produces the peak in highly viscous electrolytes. Here we can consider that, even under significant agitation, the electrode experiences a condition close to that of stagnant voltammetry with the Levich limit that is reached only at timescale larger than in water, after that a peak we the same origin of the peaks in stagnant voltammetry occurs. In the end, the peak originates from the incomplete development of the concentration profiles that happens because the high viscosity of the ionic liquid limits the renewal of electroactive species at the electrode surface.

## Conclusions

To better understand the effect of the limitation of ILs viscosity on the mass transport rate at the solid–liquid interface, we have developed a hybrid experimental/numerical approach that consists of performing RDE measurements coupled with numerical modelling. This combination allowed us to describe the extent and the time evolution of the convection boundary layer, which cannot be directly accessed experimentally. This study exploits ferrocene/ferrocinium in the BMImBF_4_ as a model system. We have observed that the high viscosity of the BMImBF_4_ dramatically limits the mass transport, even under strong mixing/stirring. We used the Levich’s analysis to calculate the diffusion coefficient of the ferrocene/ferrocinium in the BMImBF4 solvent with a good agreement with known results (Table 2). The numerical simulations of the RDE voltammetries under different conditions gave us a better insight about the nature of the voltammetric peaks, confirming that the position and extent of the peak are related to the diffusion coefficient, viscosity, scan rate and rotation speed. However, a dependence on the electron transfer kinetics of the electrolyte was also reckoned, revealing that the peak occurs under a non-reversible regime. As expected, in water, the convection layer is wholly developed for all the investigated scan rates. Conversely, for high enough scan rates, the convection layer at 470 mV (peak potential) is non-completely developed in BMImBF_4_. The analysis of the concentration profiles near the electrode revealed the connection between the emerging of the advection regime with the incomplete development of the boundary layer, and the related transport regime (convective regime). This confirms that the peaks in the voltammograms are related to the scarce provision of the species induced by an incompletely developed advection regime, anyway inadequate to restore the lack caused by red-ox process occurring at the electrode surface. Thus, the present numerical process successfully describes the occurrence of these peaks under hydrodynamic conditions in ILs and water.

This study points out also the extreme reliability of our FEA approach for the simulation of the mass transport phenomena in ILs. We have proved that the combination of RDE models and experiments is a useful tool to address the optimization of the chemical and electrochemical processes that happen in highly viscous liquids. Morever, the matching the experimental and simulated curves has enabled the estimation of ${E}_{0}$ and ${j}_{0}$. Besides, the approach can be a practical alternative the determination of the diffusion coefficient (D) and the resistance of the electrolyte ($R$).

In conclusion, the high viscosity of the ILs hinders the complete development of the advection controlled flux; consequently, the mass transport regime is under prevalent diffusion or advection control according to the scan rates and potential. On this ground, we can conclude that it is much more challenging to reach an efficient provision of the species at the electrochemical interfaces compared to water-based electrolytes. Accordingly, a special care should be taken when designing chemical and electrochemical processes operating in ILs.

## Supplementary information

Supplementary information
