# Supplementary material for: Analysis of mass transport in ionic liquids: a rotating disk electrode approach
Source: Sci Rep. 2020 Aug 10;10:13433. doi: 10.1038/s41598-020-70301-w (PMC7417597; doi:10.1038/s41598-020-70301-w)
Supplement: Supplementary file 1 — Supplementary information [file 41598_2020_70301_MOESM1_ESM.docx]

# **Electronic Supplementary Information to Analysis of Mass Transport in Ionic Liquids: a Rotating Disk Electrode Approach**

A.Giaccherini ^1^, M. Al Khatib ^2^, S.Cinotti ^1^_,_ E. Piciollo ^3^, E. Berretti ^4^, P. Giusti ^5^, M.Innocenti ^1,4^, G. Montegrossi ^6^, A. Lavacchi ^4,*^

*^1^ Dipartimento di Chimica, Università degli Studi di Firenze, via della Lastruccia 3, 50019, Sesto Fiorentino (FI), Italy.*

*^2^ Dipartimento di Biotecnologia, chimica e farmacia, Università degli Studi di Siena, via Aldo Moro,2, 53100, Siena, Italy.*

*^3^ LEM s.r.l. Via Leo Valiani, 55/59, 52025 Levane Bucine (AR), Italy.*

*^4^ CNR, Istituto per la Chimica dei Composti Organometallici (ICCOM), via Madonna del Piano 10, 50019, Sesto Fiorentino (FI), Italy.*

*^5^ CDR s.r.l., Via degli Artigiani, 6, 50055 Ginestra Fiorentina (FI), Italy.*

*^6^ CNR, Istituto di Geoscienze e Georisorse (IGG), Via La Pira 4, 50121, Firenze (FI), Italy.*

*^*^ corresponding author: alessandro.lavacchi@iccom.cnr.it*

| **Processor** | **RAM** | **Frequency** | **Computation time 1 CV** |
| --- | --- | --- | --- |
| Intel XEON E5 2660 | 64 gb ECC DDR3 | 2.2 GHz | 30-60 seconds |
| **Algebraic residual** | **Type of solver** | **Non-linear solver** | **Number of iteration** |
| <1 10^-8^ | Direct(PARDISO) | Newton( damping factor 1) | 5 |
| **Mesh type** | **Max element** | **Growth rate** | **Number of elements** |
| 1D geometric progession | 1 10^-5^ mm | 1.08 | 10000 |

**Table S1**

**

Figure S1** Simulated voltammetry of ferrocyanide oxidation in water

**Figure S2** Levich’s law applied to the linear regression of $i_{L}$ plotted against $\Omega^{1/2}$
